# Effects of Inlet Capillary Temperature in Atmospheric-Pressure Infrared Laser-Ablation Plasma Postionization Mass Spectrometry

**DOI:** 10.1021/jasms.5c00243

**Published:** 2025-10-28

**Authors:** Lilian Ellis-Gibbings, Rory T. Steven, Alex J. Dexter, Josephine Bunch

**Affiliations:** † National Physical Laboratory, Hampton Road, Teddington, Middlesex TW11 0LW, U.K.; ‡ Imperial College London, Department of Metabolism, Digestion and Reproduction, Hammersmith Hospital, Du Cane Road, London W12 0NN, U.K.

## Abstract

Mass spectrometry imaging (MSI) can be used to survey numerous molecular species from a wide variety of surfaces, including biological tissue sections. Atmospheric-pressure (AP) infrared laser-ablation plasma postionization (IR-PPI) has recently been shown to allow matrix free analysis of small molecules from both fresh frozen and formalin fixed paraffin embedded (FFPE) tissue. Detected ion intensities in IR-PPI as well as other AP inlet modalities such as desorption electrospray ionization (DESI) show a strong dependence on the inlet capillary temperature. In this study, the relationship between detected ion intensity and inlet capillary temperature is evaluated, between room temperature and 650 °C, for analyte pipetted on various substrates, as well as fresh frozen and FFPE tissue, by IR-PPI. Temperature trends for exemplar ions of interest show a variety of dependencies with optimal temperatures observed throughout this temperature range. For example, detection of lactate [M-H]^−^ *m*/*z* 89.0244 is optimal at ∼100 °C, glutamine [M-H]^−^ *m*/*z* 145.0618 at ∼250 °C, arachidonic acid [M-H]^−^ *m*/*z* 303.2324 at ∼150 °C and PI­(18:0/20:4) [M-H]^−^ *m/*z 885.5488 at ∼500 °C. Data reduction and clustering of these data by uniform manifold approximation and projection (UMAP) and k-means provides a summary of all temperature trends within the data and association of different ions with these trends are presented. Finally, the implications of different inlet capillary temperature settings in tissue MSI are demonstrated by comparing detected glucose and lactate ion intensities in response to different inlet temperatures in mouse brain. The choice and control of inlet temperature are shown to be critical variables for the interpretation of biological MSI data in AP modalities.

## Graphical abstract

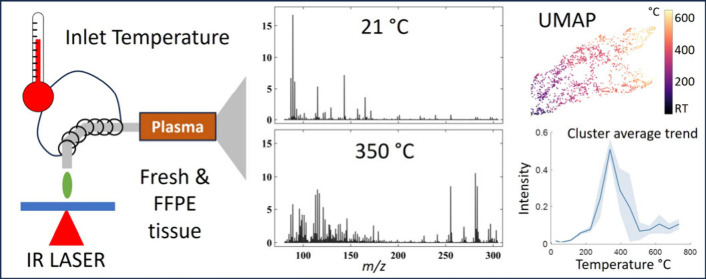

## Introduction

Mass spectrometry imaging (MSI) provides spatially resolved detection of molecular and atomic ions from a wide variety of surfaces, including biological tissues. MSI is increasingly used as a core technique in preclinical metabolomics studies alongside other spatial omics technologies such as genomics and transcriptomics. Ambient mass spectrometry relates to instruments within which the sample is held under ambient atmospheric conditions (standard laboratory temperature, pressure, and humidity) during analysis.

A wide variety of modalities exist under the umbrella of ambient MSI.^1–4^ In some cases, for improved detection of ions of interest, a form of postionization is applied.^5–7^ One such method utilizes a plasma device, known as plasma postionization (PPI). Atmospheric pressure infrared laser ablation plasma postionization MSI (AP-IR-LA-PPI MSI), referred to herein as IR-PPI,^8^ is a technique for ambient surface analysis of a range of tissue targets. Our introduction^8^ of a PPI system using a commercial, in-line, dielectric barrier discharge device as the plasma source showed several advantages over previous plasma-jet iterations of similar platforms including flexibility of mass spectrometer choice, ease of installation and minimal optimization requirements.^9,10^ IR-PPI has been used for matrix-free imaging of fresh and formalin fixed paraffin embedded tissue sections,^11^ as well as offering complementary molecular coverage to other MSI techniques.^11^ Similar IR-PPI experiments indicate nonpolar compounds are greatly enhanced by the use of plasma postionization.^12^ The use of PPI coupled to UV lasers, with and without matrix assistance, has proven useful for detection of protonated lipids^8^ and several important plant metabolites.^13,14^ Visible light (532 nm) in a similar PPI setup imaged plant tissue,^15^ while the application of fiber-optic laser delivery in the same lab allowed for a pixel size of ∼5 μm.^16^

Inlet conditions, including temperature, have been a subject of study for the optimization of successful ion transmission in ambient ionization methods and interfaces for over a decade, e.g. in solvent assisted systems.^17^ These conditions also appear critical in ion sources that permit analysis of solid samples, e.g. in UV PPI MSI,^9^ where an “optimal” experimental condition to detect lipid ions included an inlet temperature range between 270 and 400 °C.

Detection of proteins and peptides from tissue with DESI was greatly enhanced by introduction of a heated inlet capillary.^18^ Protein unfolding can even be controlled by inlet capillary temperatures, demonstrated in a DESI study^19^ on pipetted protein standards. The unfolding and denaturing of hemoglobin was controlled through inlet temperature changes between 25 and 460 °C, with dramatic spectral differences seen above 100 °C and again above 350 °C.^19^ High temperatures are well-known to be integral to other ambient mass spectrometry techniques, for example in atmospheric-pressure solids analysis probe, where a stream of hot nitrogen gas (350–500 °C) is used for the volatilization of solid samples.^20^ In fact, heated inlets alone are known to produce appreciable ion counts without the use of lasers or any other traditional ionization methods.^21^

In this study, we explore the effect of temperature in IR-PPI, for a range of analyte classes. We acquired data, in positive and negative ion modes, from fresh frozen (FF) bovine brain homogenate (BBH), FF murine brain, and formalin fixed paraffin embedded (FFPE) murine liver tissues, with an inlet capillary temperature range from room temperature to 650 °C. Statistical analysis of these data, including uniform manifold approximation and projection (UMAP) and K-means clustering, indicates global trends in the various temperature dependencies, suggesting the opportunity to enhance signal from different ions of interest through careful inlet temperature selection.

## Materials and Methods

### Materials

Mouse livers were collected from the mice following termination and then fixed in 10% neutral buffered formalin overnight. Fixed organs were placed in labeled cassettes and processed (Leica, ASP300S). Tissues were dehydrated through graded alcohols, cleared, and infiltrated with paraffin wax. Tissues were then removed from the tissue processor and embedded in paraffin wax blocks. FFPE tissues were sectioned at a 5 μm thickness. Mouse brains were collected from the mice following termination and flash frozen. Bovine brain was sourced from the human food chain. Bovine brain homogenate (BBH) was created by the following method:

BBH was produced by methods similar to those in previous work.^7^ Bovine brain (human food chain) was cut into roughly 1 cm pieces, homogenized using a high shear mixer (L4RT, Silverson Machines Inc., MA, USA) until visually uniform, and then portioned into 15 mL falcon tubes. These were centrifuged for ∼20 min (Eppendorf Centrifuge 5430R) and frozen and stored in a −80 °C freezer until sectioned.

FF BBH, whole mouse brain, and 10% gelatin blocks were sectioned at 10 μm thickness using a CryoStar MX70 cryo-microtome (ThermoFisher Scientific, USA). All tissues were mounted on standard glass slides (Thermo, Superfrost) and analyzed without further sample preparation. All animals and tissue were managed in accordance with the UK Home Office Animals (Scientific Procedures) Act 1986.

Methanol optima ≥99.9% was used as received from Fisher Scientific (UK). Glycerol ≥99.5% and glutamine 99% were used as received from Merck Life Science (UK). A 10% solution of gelatin from porcine skin, sterile, type A, from Merck Life Science (UK) was stirred at 80 °C for 10 min, set in a block mold over dry ice, and stored at −80 °C prior to sectioning.

Glutamine solution was prepared at 8.5 nmol/L in 70% MeOH:DI H_2_O v:v and 0.2 μL pipetted onto standard superfrost microscope slides coated with various substrates (bare, graphite, sectioned BBH, 10% gelatin) and allowed to dry within 1 day of measurements. Glycerol and glutamine solution comprised of 4.5 nmol/L of glutamine in 15:15:70 Glyc:DI H_2_O:MeOH v:v was pipetted on clean glass slides. Glycerol matrix spraying over analyte droplets on clean glass was carried out on a TM-sprayer (HTX Imaging, Chapel Hill, NC) prior to imaging.

### Ion Source Configuration

Details of the ion source construction and setup are provided in previous publications.^8,11^ A brief description is provided here. An infrared laser (NT377, Ekspla, LT) operated at 3 μm wavelength, with 5 ns pulse width, 20 Hz repetition rate, and approximately 1 mJ/pulse irradiated the sample, in transmission geometry, held on a microscope stage (Märzhäuser Wetzlar, DE). Ablated material entered a heated steel inlet capillary coupled to plasma device (Sicrit 15 kHz, 1.5 kV, Plasmion, DE). To heat the capillary, a resistive wire with a standard laboratory power supply (Pro Bench, RS Components, UK) was controlled and monitored by a proportional integral derivative (PID) controller and k-type S3 thermocouple (5SRTC-TT-KI-30-2M, Omega Engineering Ltd.). The resistive wire (NI80, Omega Engineering Ltd.) was electrically insulated with heat-resistant cement (Omegabond 600, Omega Engineering Ltd., Manchester, UK). Ablation tracks in fresh frozen kidney show approximately 80 μm width (Figure S1).

### Experimental Details

The ion source was coupled to an Orbitrap Elite instrument (Thermo Scientific, Germany). For all experiments the Orbitrap maximum injection time was set to 500 ms, automatic gain control off, software set mass resolving power of 60,000 (at *m*/*z* 400) in both positive and negative ion mode. Data were collected over two *m*/*z* ranges, *m*/*z* 80–305 and *m*/*z* 300–1000, to improve the measured ion counts in each *m*/*z* range in the orbitrap detector.

For analysis of pipetted glutamine on substrates, regions of interest were defined around pipetted droplets and sampled at 150, 250, 350, 450, and 600 °C inlet temperature, in random temperature order. Two pipetted droplets were included in each temperature dependent measurement in a rectangular region of interest, including pixels outside the pipetted droplet region. These regions of interest were separated into sample and background pixels via k-means clustering and analyzed separately.

Capillary temperature studies were carried out by acquiring “control” (laser off) and “tissue” data (laser on, rastering for 2 mm) for each *m*/*z* range, at each temperature setting, in both polarities for FF tissue and negative polarity for FFPE tissue. The PID was set to the desired temperature and data acquired after the measured temperature had settled for approximately 5 min at each temperature value. Data were collected at temperatures in the following order for each tissue and polarity: room temperature and 100, 200, 300, 400, 500, 600, 650, 550, 450, 350, 250, 150, and 50 °C. In the case of positive ion FF study only, the temperature values for the laser off measurement were 100, 200, 300, 400, 500, 600, 650, 550, 450, 350, and 250 °C. Measurements in positive ion mode were not taken at room temperature and 50 and 150 °C.

Imaging data were collected with flyback raster stage movement with velocity of 0.11 mm/s such that the Orbitrap scan time of 1.36 s would result in a pixel size of approximately 150 μm in the x-dimension. The stage line to line raster spacing was set to 150 μm, thus creating ∼150 × 150 μm pixels. Each new experiment and resulting.raw file, therefore, corresponds to a single image raster line. As detailed previously,^11^ the imaging procedure was controlled using an Arduino Uno microcontroller (Arduino.cc) using the start/stop signals of the stage controller (TANGO Desktop) programmed by the Switchboard software (Märzhäuser Wetzlar) to trigger laser shutters and the Orbitrap data acquisition for each new raster line.

### Data Processing

Data were first converted from Raw to mzML file formats using ProteoWizard,^22^ and to imzML using the imzML converter.^23^ Following this, datacubes of the (maximum, to capture as many real peaks as possible) top 1500 peaks were created in SpectralAnalysis^24^ by peak picking the mean spectrum after preprocessing using an interpolation-rebin zero-filling with bin size 0.00001. Datacubes were then exported to Matlab workspace, and all subsequent processing was performed using custom scripts. All presented mass spectra are the mean of 20 pixels at a constant inlet temperature.

Prior to multivariate analysis, each *m*/*z* channel was independently normalized to be between 0 and 1 to provide an equal contribution of each *m*/*z* to the overall reduced embedding and not skew the results to the highest intensity ions. UMAP was then performed on the peaks as per the workflow employed by Steven et al.^25^ using the implementation by Meehan et al.^26^ with the cosine distance metric. K-means clustering was then performed on the UMAP reduced data for a range of K values from 2 to 10 using the Euclidean distance metric and 5 replicates. The mean and standard deviation intensities for each cluster were then plotted at different temperatures, and the results from the most informative cluster number are presented.

For data evaluation and comparison of spectra acquired from laser “on” or “off” experiments, the *m*/*z* channels between the two data sets were first matched. The *m*/*z* from laser on were selected as a reference, and each peak was matched to all others in the corresponding laser off data, within a 10 ppm window. A maximum mass accuracy deviation of 5 ppm was permitted. Where multiple peaks were matched within the 5 ppm window, the intensities of those peaks were summed. Following peak matching, the mean intensity for each ion was calculated and plotted. All colors for graphs and clusters were selected using the colorbrewer^27^ tool to ensure optimal compatibility of viewing for different scenarios.

For the selection of ions to display a temperature optimum, the laser “on” PPI experimental data set was matched (to 5 ppm) to the HMDB annotations list from the Metaspace engine,^28^ the matches were subset to include only those with a false discovery rate of 10% or less, and then sorted by the metabolite signal match score. Matched *m*/*z* temperature dependent behavior was then compared between laser “on” and “off” experiments, including only those ions which show deviation from the control experiment. High scoring matches (>0.6) with annotations in the HMDB endogenous and lipidMaps lists were included. In cases of multiple isomer and isobar matches, the first listed match in HMDB is named in this paper. Additionally, several ions of interest were investigated in similar studies on IR-PPI^11^ and DESI^25^ and are included here as well for comparison, even in cases where the metabolite signal match score is lower than 0.6. Cases where an ion was previously identified in this system by MSMS^11^ are marked in the table provided. The determined matches are predominantly [M+H]^+^ and [M-H]^−^, and as such, the putative adduct is not explicitly named in the main body of the article unless the adduct differs. Stated molecule identities are tentative based on accurate mass matching and Metaspace annotation only with no additional validation. *m*/*z* values are repeated throughout as the primary indicator of ion identity.

## Results and Discussion

### Study of Pipetted Standards and Substrates

The influence of the ion source inlet capillary temperature on the detected ion intensities in IR-PPI was studied for the analysis of a pipetted glutamine standard, FFPE and fresh frozen tissue. Whether the environment and the source capillary inlet temperature are decoupled was first investigated. Glutamine was pipetted onto a range of substrates and analyzed using IR-PPI across a range of inlet temperatures. Varying the substrate allowed for an assessment of whether the temperature dependence is determined by the physicochemical properties of the analyte itself, rather than the analyte-plume environment as ejected material passes through the inlet. Additionally, this served as a compatibility test for suitable substrates for use with the IR laser system.

Glutamine solution was deposited onto glass slides, sectioned BBH, sectioned gelatin, a thin layer of graphite, additionally codeposited with glycerol, and analyzed with the IR-PPI setup. Relevant ion intensity vs inlet temperature plots are shown in Figure 1. Glutamine solutions pipetted directly onto glass slides did not yield appreciable metabolite ion intensities in the IR-PPI setup used here. It is reasoned that without a substance present to act as a solid-to-gas-phase carrier or “matrix” (as for matrix assisted laser desorption ionization, MALDI), the energy transfer is insufficient to desorb/ablate significant glutamine from the glass surface. Optical inspection of the sample failed to support or refute this potential level of ablation due to the lack of contrast for pure standard pipets on glass. Future studies characterizing this would be of interest. In contrast, droplets deposited on top of both sectioned BBH and sectioned gelatin yielded a high intensity for the [M-H]^−^ glutamine peak. Graphite has been used as a substrate to enhance laser desorption in SALDI-MS,^29^ however no appreciable ion signal was detected from droplets on graphite here. It was noted that the droplets on graphite were subject to increased wetting, causing unwanted droplet spreading while drying. Glycerol can be used as an IR-absorbing matrix,^30^ however spraying a glycerol coating on the prepipetted droplets on glass slides led to unwanted delocalization of the pipetted glutamine. Manual deposition of premixed glutamine with glycerol resulted in somewhat inconsistent droplet size as well as inconsistent ion counts detected between different droplets.

**Figure 1:**
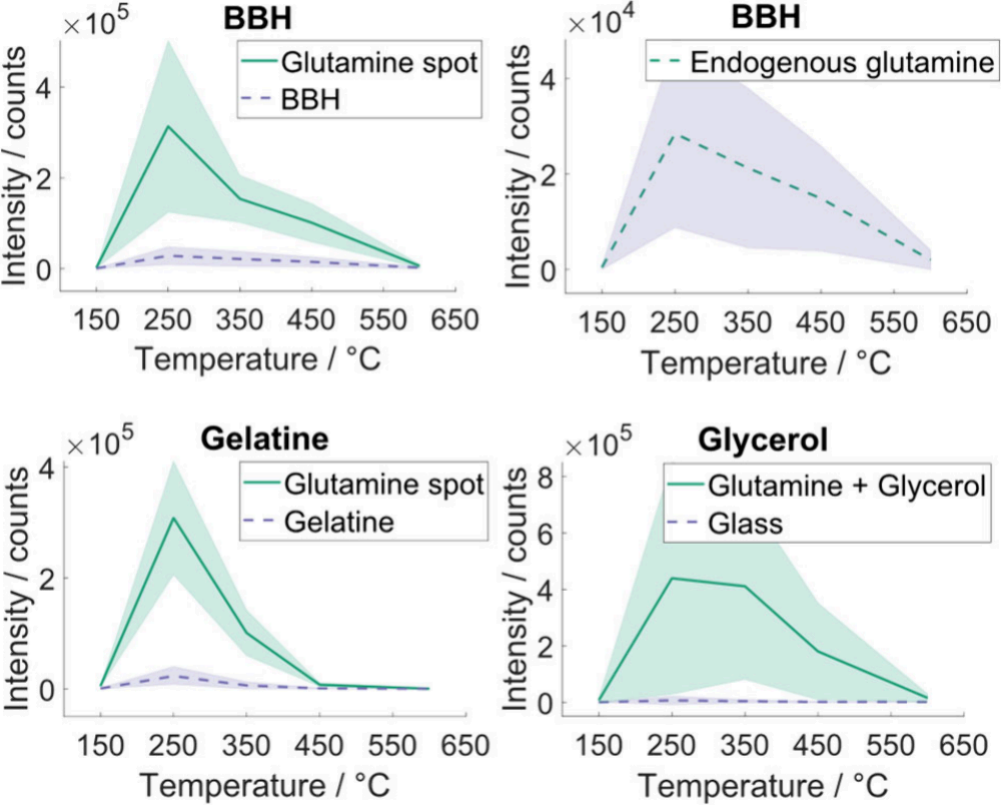
Ion intensity response of glutamine [M-H]^−^ (*m*/*z* 145.0618) to temperature for different environments. Top left: deposited on BBH sectioned onto superfrost slide. Top right: endogenous glutamine in BBH. Bottom left: deposited on gelatin sectioned onto superfrost slide. Bottom right: glutamine/glycerol solution deposited on superfrost slide. Lines connect mean count data points at each temperature, shading indicates ±1 standard deviation.

In the three cases of droplets of glutamine on BBH, gelatin, and codeposition with glycerol, the intensity of the glutamine peak showed inlet temperature dependence similar to that of endogenous glutamine in a fresh frozen tissue sample (Figure 1). The temperature optima of the detected ions were therefore considered to not be (primarily) determined by the tissue environment nor, therefore, the ejected plume composition. For all substrates, the optimal inlet temperature measured was 250 °C, decreasing slowly with increasing temperature, with near baseline detection at 150 and 600 °C.

The novel use of gelatin as a sectioned substrate for pipetted standards in conjunction with IR-PPI was found to provide a good signal-to-noise ratio for measurements. Gelatin is easy to prepare and section and appears to allow for suitable energy transfer between the IR laser and the pipetted sample, enabling ejection into the gas phase.

### Study of Fresh Frozen and FFPE Tissues—Spectral Summaries

The behaviors of endogenous molecular species do not all follow the same temperature dependence as that of glutamine. Mean mass spectra from BBH across the range of inlet capillary temperatures, in negative ion mode, displayed in Figure 2, attest to this. Significant changes in the collected spectra can be seen between room temperature and 100 °C. Notably, both *m*/*z* = 89.0244 and 87.0087 increase in intensity by approximately an order of magnitude between 24 and 100 °C.

**Figure 2:**
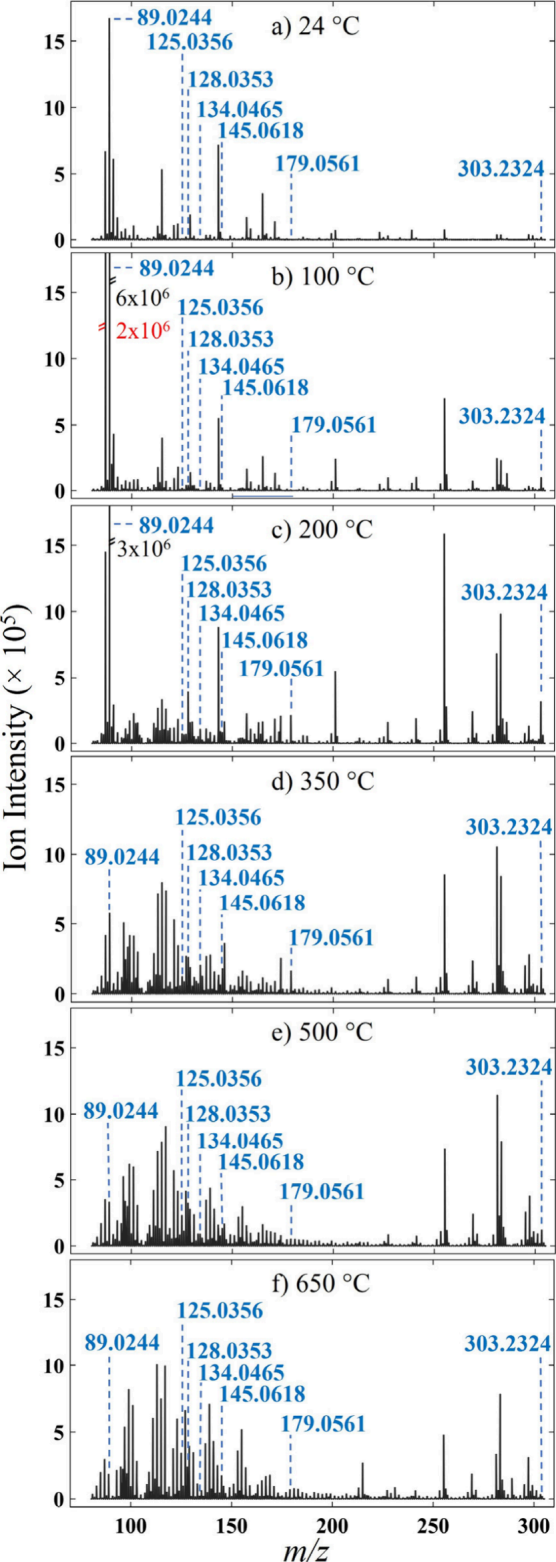
IR-PPI negative ion mode mean mass spectra from fresh frozen bovine brain homogenate at increasing capillary inlet temperatures, as denoted in (a)–(f). Double dashed lines and associated intensity values in (b) and (c) indicate intensity of corresponding peaks where intensity scales have been restricted to create matched *y*-axis ranges across all subfigures.

A broad trend for ions in the range *m*/*z* 150–305 sees an increase in detected ion intensity with temperature, with a maximum at 200 °C, before decreasing slowly as temperature is further increased (Figure 2). For ions detected at lower *m*/*z* values, e.g., *m/*z 80–150, a mixed picture is evident. An obvious increase in the number of high intensity peaks is present with increasing temperature; however, several peaks with high intensity at 24 °C reduce in intensity with increasing temperature.

Ions, assigned through Metaspace (as described), are annotated in Figure 2. These include pyruvate *m*/*z* 87.008, lactate *m*/*z* 89.0244, thymine *m/*z 125.0356, pyroglutamic acid *m/*z 128.0353, adenine *m/*z 134.0465, glutamine *m/*z 145.0618, glucose *m/*z 179.0561 and arachidonic acid *m/*z 303.2324. Peaks that constitute the “background” signal exist at every temperature measured. Their source and features are discussed in detail in the article and Supporting Information (SI) introducing this setup,^8^ and they include hotspots and carry-over expected to be caused by slow desorption from the capillary wall.

Temperature-dependent trends for *m*/*z* signal intensities with inlet temperature were seen across both ion polarities, for the *m*/*z* 80–305 mass range and the *m*/*z* 300–1000 mass range, across both fresh and formalin fixed tissues. To highlight the spectral differences, a comparison between 250 and 500 °C is made for each case. Example mean mass spectra for negative and positive ion studies on FF BBH are shown in Figure 3, while example mean mass spectra for negative ion FFPE livers are displayed in Figure 4. Significantly different spectra are obtained from the fresh BBH and FFPE liver, as expected.^11^

**Figure 3:**
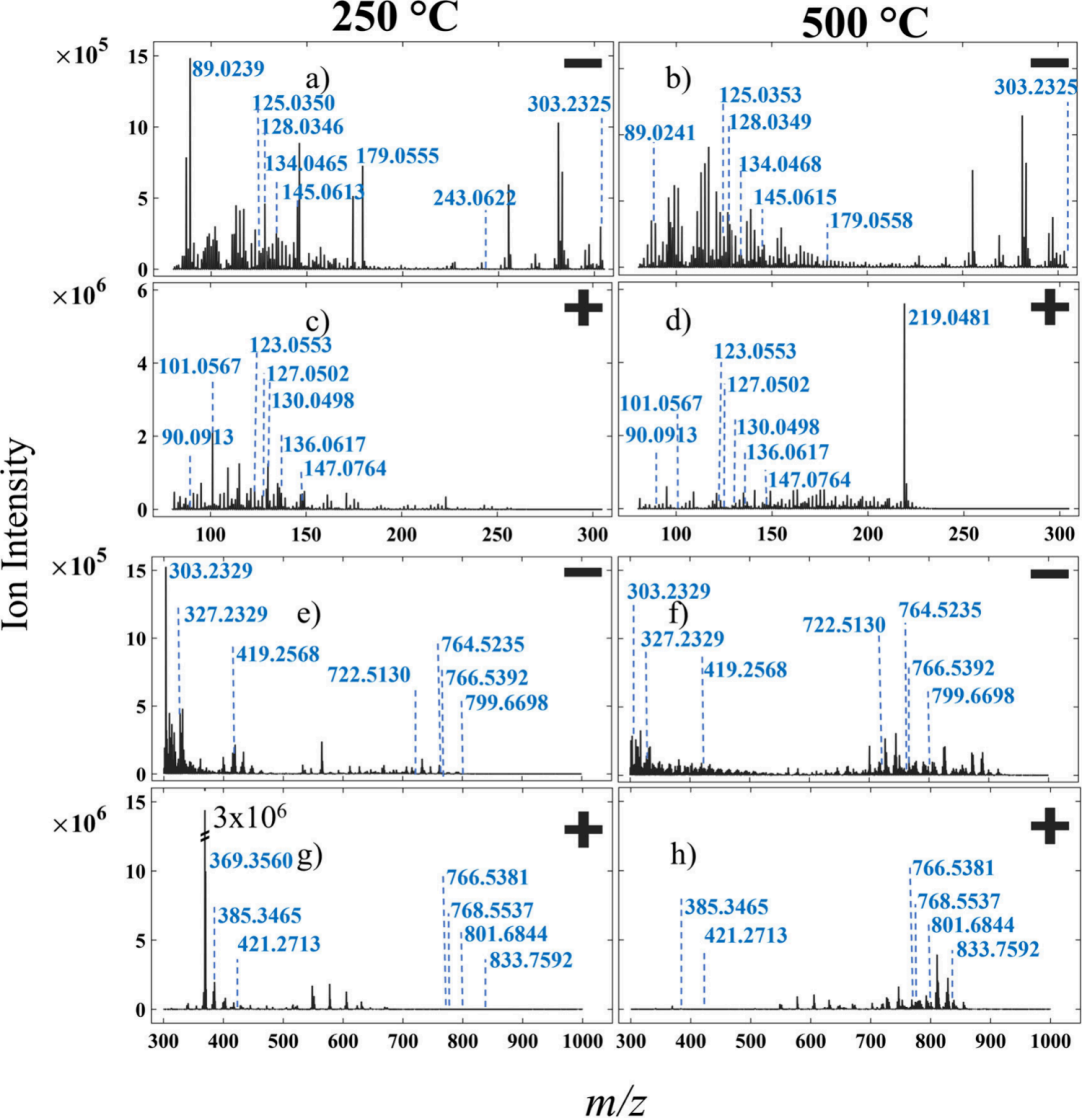
Mean mass spectra acquired from fresh frozen samples using IR-PPI MSI highlighting the differences between 250 °C (left column) and 500 °C (right column) capillary inlet temperatures, as follows. (a,b,e,f) Fresh Frozen BBH negative ion mode. (c,d,g,h) Fresh Frozen BBH positive ion mode. *m/z* ranges are *m*/*z* 80–305 (a–d) and 300–1000 (e–h).

**Figure 4:**
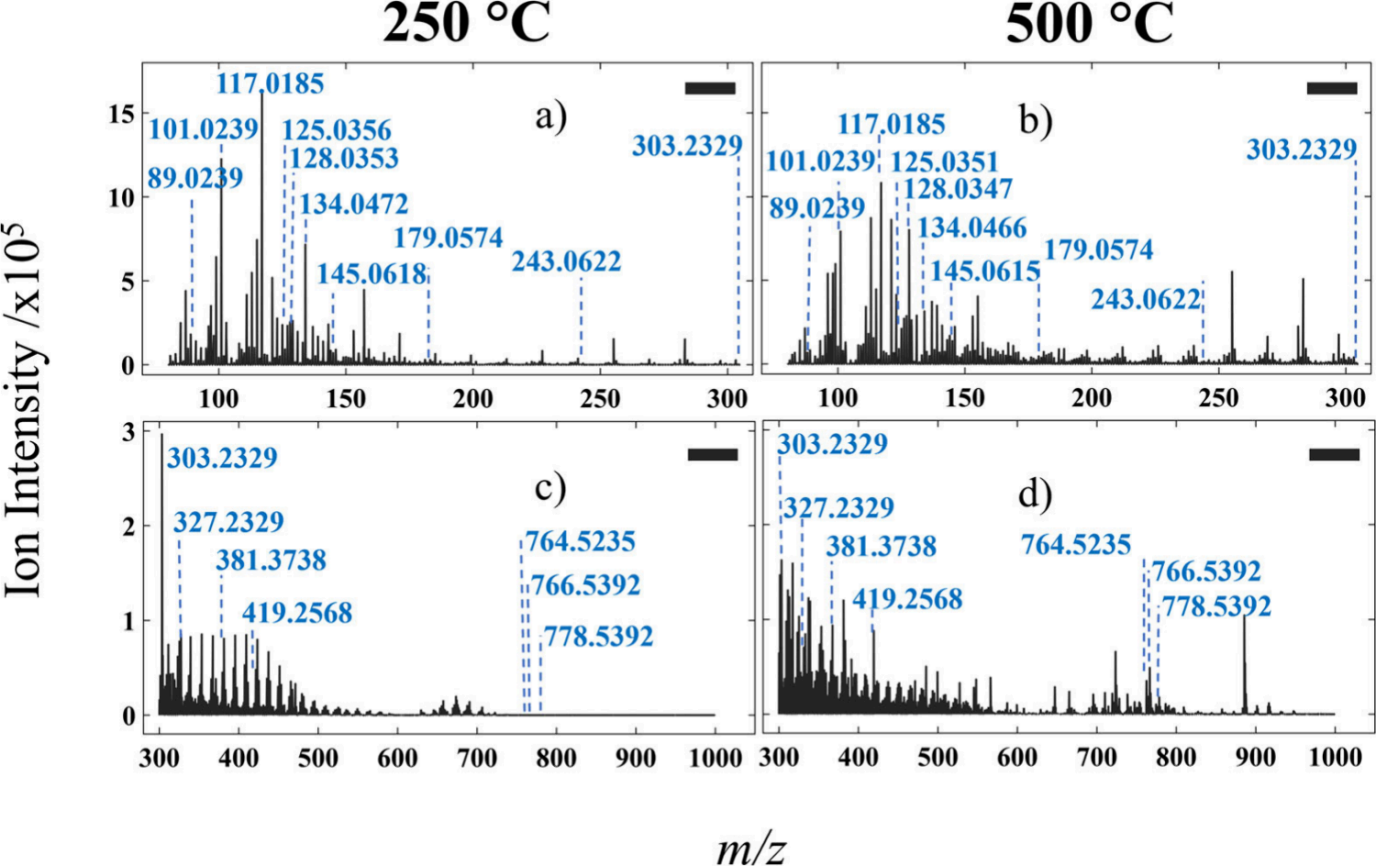
Mean negative ion mass spectra acquired from formalin fixed paraffin embedded samples using IR-PPI highlighting the differences between 250 °C (left column) and 500 °C (right column) capillary inlet temperatures. *m/z* ranges are *m*/*z* 80–305 (a,b) and 300–1000 (c,d).

The full range of temperatures (24, 50, 100, 150, 200, 250, 300, 350, 400, 450, 500, 550, 600, and 650 °C) show a similarly large range of spectral changes as between the two temperatures shown (all mean spectra are displayed in SI Figures S2–S5). Figure 3 displays eight graphs showing mean mass spectra for two inlet temperatures, two polarities, and two mass ranges for FF BBH. Initially, in Figure 3a,c, there are a similar number of ions below *m*/*z* 180 in both negative and positive polarity. There are significantly more ions between *m*/*z* 225–305 in negative polarity compared to positive. Analagous observations can be made for the *m*/*z* 300–1000 range, where Figure 3e,f shows what appears to be a broader range of ions detected in negative polarity than Figure 3g,h. The effect of the two capillary temperatures is visible across the eight spectra. In the mass range *m*/*z* 80–305, where small metabolites are commonly detected, several prominent ions in Figure 3a,c are much reduced in the higher temperature data (Figure 3b,d). When comparing 250 to 500 °C for positive polarity (Figure 3c,d) a prominent new base peak at *m/*z 219.0481 (unassigned to 5 ppm error in HMDB via Metaspace), as well as a broader range of ions between approximately *m*/*z* 150 and 220, appear at the higher temperature. There is also a clear increase in the peak intensities between *m*/*z* 700–900 at 500 °C in both polarities (Figure 3f,h), indicating an increase in lipids such as PC (35:4) *m*/*z* 766.5381. The decrease in peak intensities at 500 °C between *m*/*z* 300–650 is present in negative polarity for peaks such as docosahexaenoic acid^31^ *m/*z 327.2329 and CPA(18:0)^32^ *m*/*z* 419.2568. This same decrease is even more striking for the positive polarity measurement (Figure 3f,h) in the *m*/*z* 300–650 range, including the complete reduction of the base peak at *m*/*z* 369.3560.

The detected base peak *m/*z varies across polarities, mass ranges, and inlet temperatures for fresh tissue. In negative polarity, the spectrum presented in Figure 3a has a base peak of lactate *m/*z 89.0239, for the 250 °C inlet temperature, and arachidonic acid *m*/*z* 303.2325, at 500 °C (Figure 3b). In positive ion mode, Figure 3c,d, the base peak ions detected at *m*/*z* 101.0567 and *m*/*z* 219.0481 were unassigned in the HMDB via Metaspace. Figure 3e,f features negative polarity base peaks of arachidonic acid^11^ *m*/*z* 303.2327 and *m*/*z* 317.2126, possible fatty acid related metabolite (for example^33^ HETE, leukotriene A4), at 250 and 500 °C inlet temperature, respectively. In positive ion mode above *m*/*z* 300, the base peak at 250 °C, *m*/*z* 369.3502, exceeds the intensity of the remaining ions by an order of magnitude (Figure 3g). This ion has been previously identified as the protonated water loss species of cholesterol^7^ [M-H_2_O+H]^+^. At 500 °C, Figure 3h, the positive ion base peak is at *m*/*z* 810.6885, assigned as either glucosyl- or galactosyl-ceramide^7^ (d18:1/24:1­(15Z)).

The four graphs displayed in Figure 4 show the varied IR-PPI mass spectra at two inlet temperatures, two polarities, and two mass ranges for FFPE murine liver tissue. Small changes of ion intensity increases and decreases are seen when changing from 250 to 500 °C for the mass range *m*/*z* 80–175 (Figure 4a,b), examples being the rise of pyroglutamic acid *m*/*z* 128.0346 and the fall of ketobutyric acid *m*/*z* 101.0244. Above approximately *m*/*z* 175, the number and intensity of ions notably increases (Figure 4b); however, few of these are assigned via Metaspace. The changes between 250 and 500 °C are stark above *m*/*z* 300 (Figure 4c,d). A clear rise in lipid signal is seen at 500 °C; for example, PC(36:5) *m*/*z* 778.5392 (Figure 4d) as well as a broad range of *m*/*z* peaks between 300 and 419, including a lower intensity arachidonic acid *m*/*z* 303.2324 and a higher intensity CPA(18:0) *m*/*z* 419.2568 (Figure 4c,d).

Unlike in FF BBH, the base peak in spectra collected from the FFPE sample remained the same *m*/*z* across both temperatures, as seen in Figure 4a–d. Base peaks in FFPE are succinic acid *m*/*z* 117.0185 in the *m*/*z* 80–305 mass range and arachidonic acid *m*/*z* 303.2327 in the *m*/*z* 300–1000 mass range at both temperatures shown.

There is a pattern of repeating peaks between approximately *m*/*z* 300 and 500, tentatively assigned here to paraffin polymer ions with differing base unit number (Figure 4). Paraffin hydrocarbons thermally decompose into carbon chains of saturated and unsaturated hydrocarbons, and olefins, of varying lengths.^34^ At higher inlet capillary temperatures (Figure 4d), these peaks are present but less prominent, indicating their suppression or alteration through temperature effects including possible thermal decomposition.

### Study of Fresh Frozen and FFPE Tissues—Temperature Graphs

From the mass spectrometry studies producing data shown in Figures 2, 3, and 4 and SI Figures S1, S2, S3, and S4, the temperature-dependent ion intensity behavior of individual ions in tissue can also be explored. The temperature trends of several ions of interest are displayed in Figure 5, from FF BBH in negative and positive ion modes and FFPE murine liver in negative ion mode. As noted, in our IR-PPI apparatus, very few ions arising from tissue show detected intensities above the background at inlet capillary temperatures below 100 °C, notably aside from lactate *m*/*z* 89.0239 (Figure 5a). Several detected ions show a clear optimal temperature in the studied range, with examples shown in Figure 5. Ions with lower *m*/*z* often show optimal temperatures in the range 100–300 °C. Examples of this behavior are seen in Figure 5 for *m*/*z* 89.0241 (a), 89.0239 (d), 90.0193 (b), 125.0348 (d), 145.0614 (a), 303.2327 (a), 327.2332 (a), and 381.3782 (d). The higher *m/*z signals of lipids generally exhibit narrower optima centered around higher temperatures, e.g. at *m*/*z* 722.5174 (a), 766.5432 (a), and 885.5490 (d).

**Figure 5:**
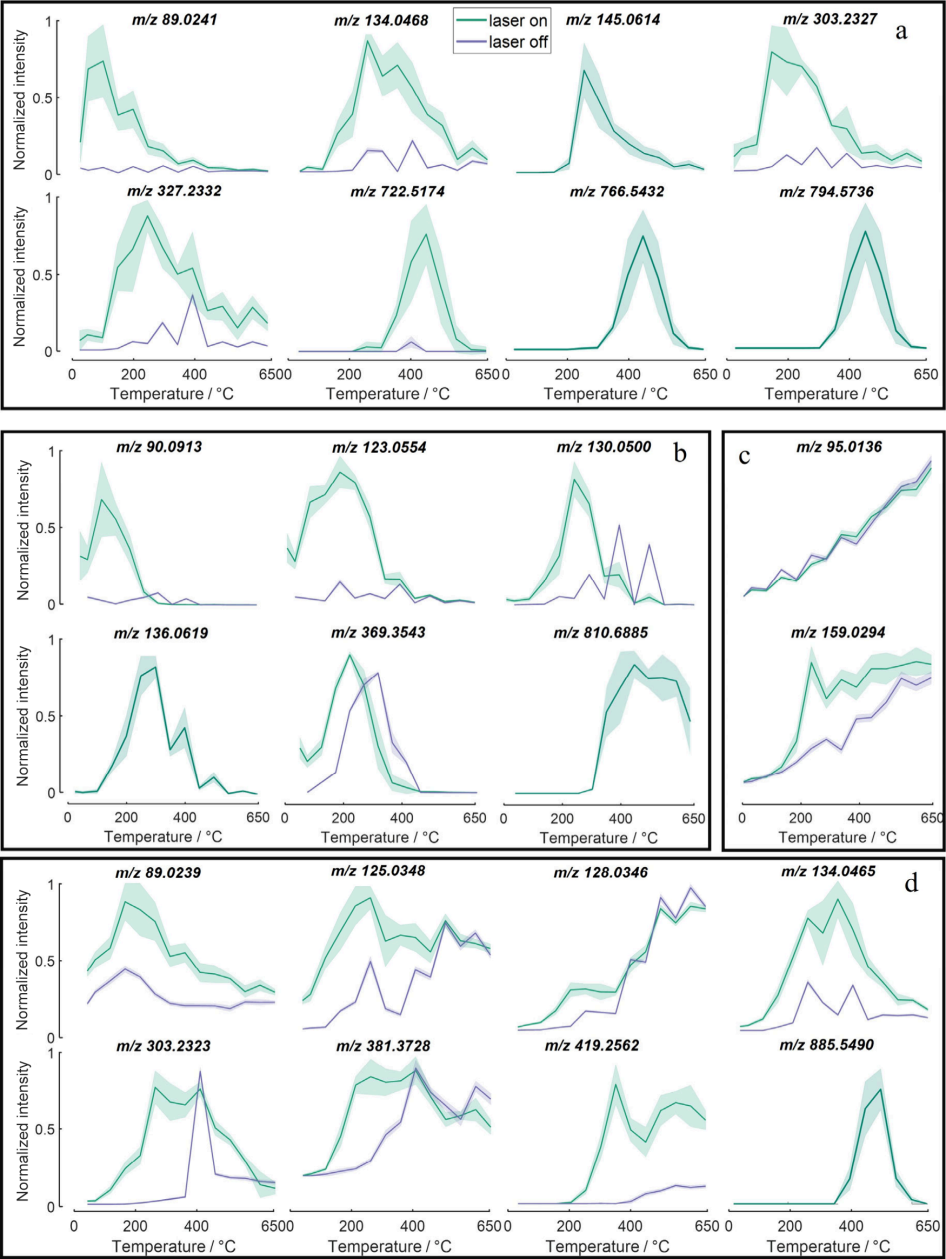
Ion intensity vs temperature for a selection of ions detected in FF BBH and FFPE murine liver (green). Laser off control data are shown in purple where a signal for the same *m*/*z* is detected. Lines connect mean count data points at each temperature (24–650 °C); shading indicates ±1 standard deviation. (a) FF BBH negative ion mode, (b) FF BBH positive ion mode, (c) FF BBH negative ion mode, where background and signal rise concomitantly with temperature, (d) FFPE murine liver negative ion mode. Ion *m*/*z* indicated on individual plots.

A paper by Michael et al.,^9^ utilizing a heated inlet/Sicrit device configuration similar to that in our lab, interfaced with a Bruker TimsTOF fleX, using a 355 nm laser on liver tissue homogenate, studied the effect of inlet temperature on lipid ion intensity. In their study, optimal temperatures for lipid analysis were found between approximately 200 and 350 °C. The authors provide class by class optima by averaging 5 *m*/*z* signals from each class, and so some direct comparisons can be made. Notably, the lipids shown to have the lowest optimal temperature are the least readily detected in our study, being, for example, DG, diox, and TG. Ceramides are detected in both works, though in the Michael et al. study, optimal temperatures for ceramide detection were at 250 °C vs 450 °C in the current work. In the case of the phospholipid PE, the UV-PPI work has an optimal inlet temperature of approximately 400 °C, whereas with IR-PPI the optimum for PE­(18:3/P-18:1) is 450 °C. For PC and PA, Michael et al. show a consistently increasing signal up to 500 °C, where their measurements end. In our work, the optima for several PC and PA species are 400 and 450 °C, lower than these values. These differences of up to 200 °C in optimal temperature may arise from differences in laser wavelength, inlet construction, temperature monitoring, and ion block/MS interface temperature and pressure differential. Regarding laser wavelength, if the initial plume properties and molecule internal energies are altered, potentially different optimal temperatures will be observed, as noted by Michael et al. in their discussion.

The clear influence of temperature on detected ion intensity means that for targeted approaches, ions of interest can be enhanced. For guidance, the optimal temperatures in this experimental setup for a range of ions are provided in Table 1. For the largest number of detected peaks below *m*/*z* 300, a capillary inlet temperature near 250 °C provides a balance between those ions optimized at low and high temperatures. Between *m*/*z* 300–1000, a higher inlet temperature of approximately 450 °C is expected to capture many of the ions of interest present in this range.

**Table 1:** Optimal Capillary Temperature for Selected Ions^a^

Annotation	*m*/*z*	Adduct	Tissue/form	Optimal Temp, °C
PI(38:4)	885.5488^a^	[M-H]^−^	Liver/FFPE	500
GalCer(42:1)	810.6885	[M+H]^+^	BBH/FF	450
PC(37:4)	794.5736	[M-H]^−^	BBH/FF	450
PC(36:5)	778.5392	[M-H]^−^	Liver/FFPE	400
PC(35:4)	768.5538	[M+H]^+^	BBH/FF	450
PC(35:4)	766.5392	[M-H]^−^	BBH/FF	450
PC(35:4)	766.5388	[M-H]^−^	Liver/FFPE	400
PC(33:4)	738.5079	[M-H]^−^	Liver/FFPE	400
PE(P-36:4)	722.5130	[M-H]^−^	BBH/FF	450
PA(38:6)	719.4657	[M-H]^−^	Liver/FFPE	450
CPA(18:0)	419.2568	[M-H]^−^	Liver/FFPE	350
Pentacosanoic acid	381.3738	[M-H]^−^	Liver/FFPE	250
Cholesterol	369.3543	[M-H_2_O+H]^+^	BBH/FF	200
Docosahexaenoic acid	327.2329	[M-H]^−^	BBH/FF Liver/FFPE	250
FA(20:4)	303.2329^a^	[M-H]^−^	Liver/FFPE	250
FA(20:4)	303.2325^a^	[M-H]^−^	BBH/FF	150
d-Glucose	179.0553^a^	[M-H]^−^	BBH/FF	250
Stearic acid	283.2639	[M-H]^−^	BBH/FF	200
Oxoadipic acid	159.0294	[M-H]^−^	BBH/FF	250
Glutamine	145.0618	[M-H]^−^	BBH/FF	250
Aminobenzoate	138.0553^a^	[M+H]^+^	BBH/FF	300
Adenine	136.0619	[M+H]^+^	BBH/FF	300
Hypoxanthine	135.0307	[M-H]^−^	BBH/FF	250
Adenine	134.0472	[M-H]^−^	BBH/FF Liver/FFPE	250
Pyroglutamic acid	130.0498	[M+H]^+^	BBH/FF	300
Pyroglutamic acid	128.0353	[M-H]^−^	BBH/FF, Liver/FFPE	300
Thymine	125.0356	[M-H]^−^	BBH/FF, Liver/FFPE	250
Picolinic acid	124.0392^a^	[M+H]^+^	BBH/FF	300
Niacinamide	123.0552^a^	[M+H]^+^	BBH/FF	200
Succinic acid	117.0185^a^	[M-H]^−^	Liver/FFPE	350
Ketobutyric acid	101.0244	[M-H]^−^	Liver/FFPE	250
Dimethylethanolamine	90.0913	[M+H]^+^	BBH/FF	100
Lactate	89.0244	[M-H]^−^	Liver/FFPE, BBH/FF	100
Lactate	89.0239	[M-H]^−^	Liver/FFPE	150

a In most cases the first appropriate HMDB match is included for brevity.

b Indicates ions previously assigned by MS/MS.^11^

Comparing the optimal temperature between the polarities studied in FF BBH or FFPE liver data, there are slight differences noted in the optimal temperature for select metabolites (Table 1, e.g., lactate, adenine, arachidonic acid) in contrast to the preliminary work in Figure 1. These deviations are either 50 or 100 °C, and the smallest temperature increment in our experiment is 50 °C. It is possible the real optimal values are found between these points, however other explanations for the discrepancy may exist. The likely more heterogeneous metabolite distributions in the FFPE liver tissues used here may confound the interpretation of resulting temperature trends to a degree. Though normal liver tissue can show relatively homogeneous distributions,^35^ variation due to any present heterogeneity is of course captured in the standard deviation shown with each graph (Figure 5), meaning significant differences shown are still highly relevant for temperature studies.

In Figure 5c, the behavior of ions whose intensity increases concomitantly with temperature are displayed. This is a general trend also seen across a broad range of ions not shown here. The first is those where there is no deviation between laser ‘on’ and laser ‘off’ measurements and no or only unrealistic annotations in Metaspace. These are assumed to be non-endogenous, as for *m/*z 95.0136 (Figure 5c). The second case describes ions where a discernible signal is detected on top of the rising background signal, as for oxoadipic acid *m/z* 159.0294, indicating the confluence of real ion signal over background signal (Figure 5 c). Only those ions that have a laser “off” signal lower than their laser “on” signal can be considered tissue-derived, regardless of annotation. Background signal from laboratory atmosphere and, to a lesser extent, tissue carryover are relatively well-known phenomena in plasma ionization MS. Please see previous work for further discussion on this topic.^11^ In brief, significant background signal is observed from laboratory air where the plasma device is turned on and relatively long-lived carryover can be seen within IR-PPI tissue ion images correlating with the detection of tissue endogenous species in previous pixels.

It is of note that the boiling point of paraffin wax is 370 °C,^36^ and if this wax is contaminating the inlet capillary, then any tissue cluster or layer adsorbed alongside would be released into the plasma along with the evaporating paraffin above this temperature. This hypothesis could explain the rising experiment and control signals in FFPE systems, particular examples of this behavior being for *m*/*z* 128.0346, 303.2323, 381.3728, and 419.2562 (Figure 5d).

### Study of Fresh Frozen and FFPE Tissues—UMAP and Cluster Average Trends

The effects of the inlet temperature seen across the range of mass spectra and I vs T ion trends in Figures 2–5 are part of general temperature-dependent trends throughout the MS data collected in this study. To interrogate the global effects of inlet temperature on the entire detected ion population, multivariate analyses by UMAP and k-means clustering were undertaken. Here we employ this approach for the full spectral IR-PPI inlet temperature data set for negative ion mode measurements of FF BBH (Figure 6 for *m/*z 80–305 and Figure 7 for *m*/*z* 300–1000). The distribution of *m/z*, optimal temperature, and effect size within the UMAP embedding space are also displayed.

**Figure 6:**
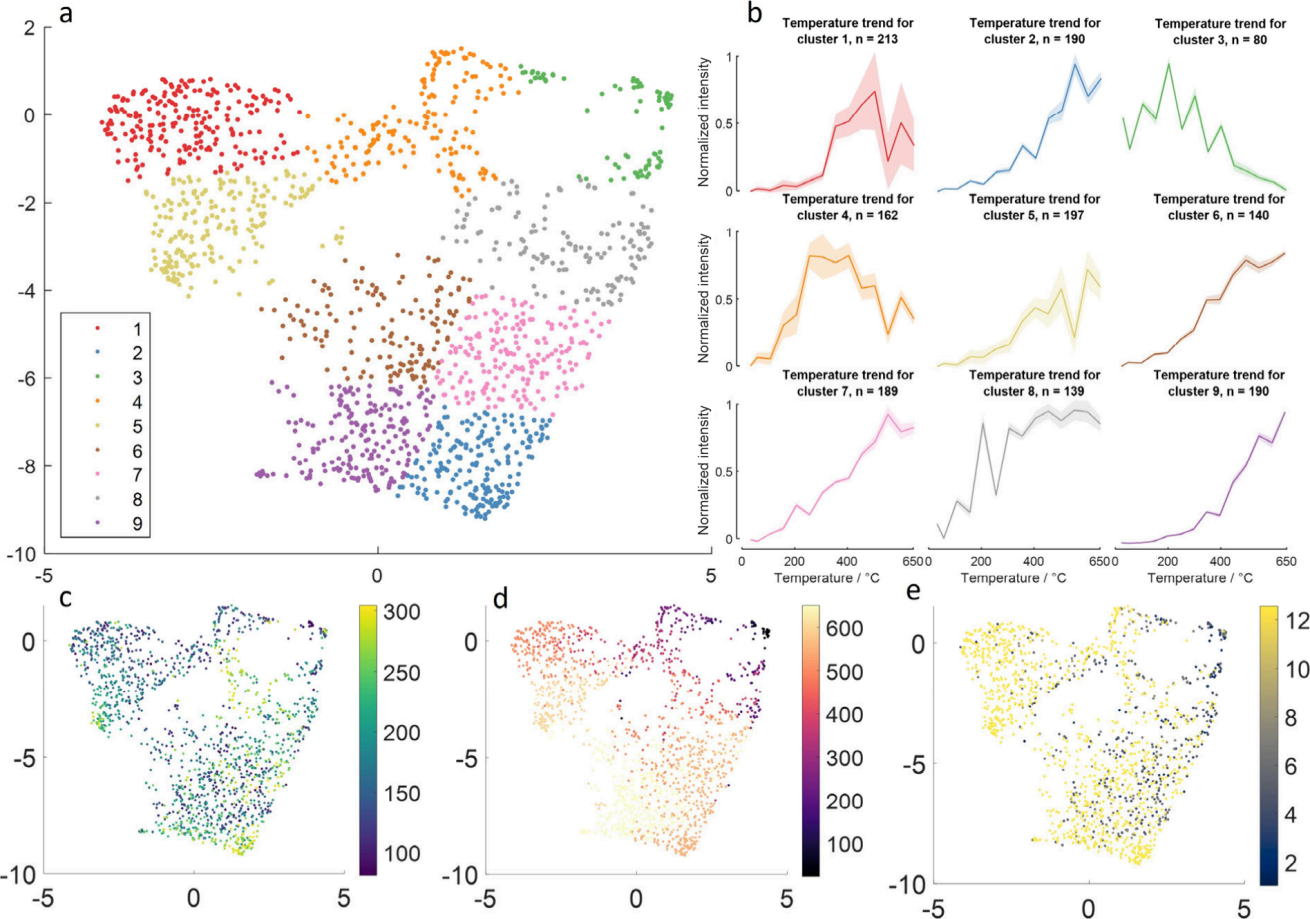
UMAP and k-means clustering across temperature trends. Negative ion mode, FF BBH, measured across *m*/*z* 80–305 on the IR-PPI system. (a) UMAP embedding clustered by k-means clustering alongside the (b) associated average intensities across the temperature ranges per cluster. The UMAP embedding is also shown labeled according to key variables including (c) *m*/*z*, (d) optimal temperature, and (e) log_2_(maximum intensity/minimum intensity).

**Figure 7:**
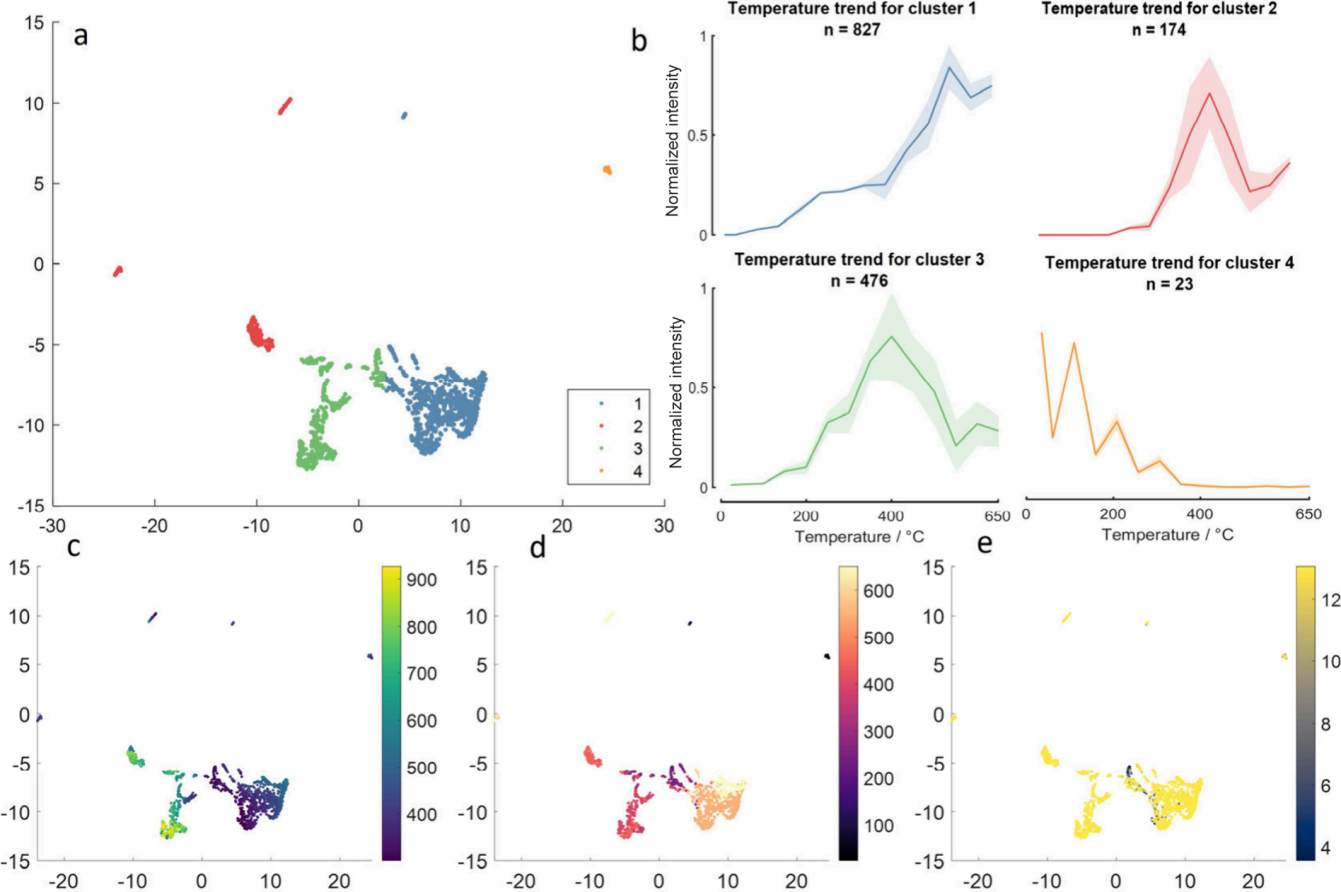
UMAP and k-means clustering across temperature trends. Negative ion mode, FF BBH, measured across *m*/*z* 300–1000 on the IR-PPI system. (a) UMAP embedding with k-means clustering alongside the (b) associated average intensities across the temperature ranges per cluster. The UMAP embedding is also shown labeled according to key variables including (c) *m*/*z*, (d) optimal temperature, and (e) log_2_(maximum intensity/minimum intensity).

The 2D UMAP embedding (Figure 6a) shows a somewhat homogeneous spread, reflecting the continuum of temperature responses across the ion population. Clusters 2, 6, 7 and 9, for example, show similar trends in Figure 6b and are not spatially separated in Figure 6a. These temperature trends (as well as those trends shown in clusters 5 and 8 in Figure 6b) show increasing ion intensity with temperature, and in fact, most detected ion signals are described within these clusters (1045 of 1500 ions). As illustrated in Figure 5c, many of these rising signals do not deviate from background measurements and are therefore not considered to be tissue endogenous. Clusters 1, 3, and 4 show different trends, each with a different optimum temperature. The distribution of ion *m*/*z* throughout the UMAP representation for the *m*/*z* 80–305 mass range data (Figure 6c) shows no clear trend. The distribution of ions across the 2D UMAP embedding correlates with the optimal temperature to detect that ion (Figure 6d), indicating that this may be the primary driver of the distribution itself. No clear trends are seen with fold change of maximum vs minimum intensity (Figure 6e). The log2­(maximum intensity/minimum intensity) is included to show the fold improvement gained by selecting an optimal inlet temperature, which in strongly influential cases is a factor of 12.

The UMAP analysis of the *m/*z 300–1000 mass range data shows a well separated representation of the clusters in the UMAP 2D space (Figure 7a). The majority of ions (827 of 1500) increase in ion intensity with temperature and thus are clustered together (cluster 1, Figure 7b). Three other apparent trends were evident from the clustering. Two clusters with single optima are seen (clusters 2 and 3) while cluster 4 decreases in intensity, containing, however, only 23 ions. A more obvious correlation of *m*/*z* with the UMAP 2D embedding is seen in Figure 7c, with the ions between *m*/*z* 300–500 most closely associated with cluster 1. This region of the mass spectrum contains peaks related to fatty acids and DAGs; however, the constantly increasing signal intensity noted in cluster 1 is often associated with background peaks, as seen in Figure 5c and associated discussion. There is a possibility, therefore, that lipid ions are clustered in clusters 2 and 3, while cluster 1 contains many nonendogenous ions. The optimal temperature for ion detection (Figure 7d) correlates well with the clusters in this mass range, again indicating that optimal temperature does drive the clustering here. Unlike in Figure 6, the intensity fold change distribution (Figure 7e), when mapped onto the 2D UMAP representation, shows distinct regional distribution with the majority of ions showing approximately a factor of 12-fold change. The lowest fold change ions are present in a tight distribution spanning the joining area of clusters 1 and 3.

Considering both the *m*/*z* 80–305 and *m/*z 300–1000 (Figures 6 and 7, respectively), the optimal temperature as mapped onto the UMAP 2D representation for each *m*/*z*, (Figure 6d and 7d) show a clear distribution which is, as expected, similar to the clustering that delineates the temperature trends (Figure 6a and 7a). For those clusters that show an optimal temperature, the optimal temperatures are between 200 and 450 °C in the *m/*z 80–350 mass range (clusters 1, 3 and 4), while the optimal temperatures in the *m/*z 300–1000 mass range (clusters 2 and 3) are between 300 and 500 °C. Figure 7d, when compared to Figure 7a,b, also indicates that many lipids in the *m*/*z* 700–800 range are clustered into two clusters: cluster 2, with a narrower optimum centered on 450 °C, and cluster 3, with a broader optimum centered on 400 °C.

Our previous research demonstrated the use of UMAP for dimensionality reduction and visualization of inlet temperature-ion intensity trends in DESI MS.^25^ That analysis showed a similar range of temperature trend profiles. However, IR-PPI exhibits proportionally larger ion populations with trends that increase with increasing temperature. Notably, at room temperature, where IR-PPI does not detect large numbers of endogenous ions, the ion intensity in DESI is high. In fact, the intensity of ions detected via DESI across a temperature range from room temperature to 500 °C may scarcely double, where detected ion intensities in IR-PPI can span multiple orders of magnitude across the same temperature change. The various temperature trends observed in DESI also vary in the population size. For example, here, in the data set acquired from *m*/*z* 300–1000, Figure 7b, cluster 4, characterized by ions exhibiting a decreasing intensity with increasing temperature, describes just 23 out of the 1500 ions (1.5%). In DESI experiments, the equivalent decreasing cluster describes 685 out of 4000 ions (17%). In contrast to IR-PPI, DESI produces significant ion counts across the *m*/*z* range without heating the inlet capillary. It is also notable that the relative fold improvement in ion intensity at optimal temperature compared to least optimal is much greater in IR-PPI compared to DESI. In an example of this trend, the ion intensity for *m/z* 89.0239, lactate, has a fold change of 17 for IR-PPI and 6 for DESI, between the optimal temperature of 100 °C and the least optimal temperature measured across both instruments, 500 °C. Most of the ions in the IR-PPI data set (e.g., 1393 out of 1500 for the *m*/*z* 300–1000 data) have intensities of zero at some temperatures, again indicating the criticality of the inlet temperature.

UMAP embedding was also performed for the data acquired from FFPE murine liver and the positive ion data from fresh frozen BBH. These are shown in the SI (Figures S6 and S7, respectively). The general ion intensity trends with the capillary temperature show very similar behavior in positive ions and in FFPE sample types. In general, there are a range of trends that show increasing ion intensity with increasing temperature as well as trends showing a maximum at an optimal temperature, while very few ions cluster into trends that decrease in intensity with temperature.

### Study of Brain Tissue in Imaging Mode

Studying the ratio of ions involved in metabolic processes can be used to infer metabolic changes in biological systems. An interesting example is the detection of relative amounts of glucose and lactate to track manifestations of the Warburg effect. Such ratios can be used to monitor the shift from oxidative phosphorylation to rapid aerobic glycolysis that goes along with a high rate of lactate production in tumors.^37,38^ Our results indicate large changes in detected intensity with inlet temperature, including ion signals that increase from zero counts to 10^5^ or 10^6^ (Figure 2), despite the fact that the endogenous concentration of the primary species in the tissue is assumed little changed, particularly in the case of homogenate.

As inlet capillary temperature optima in ambient MSI vary for different ion species, the relative intensities are thus also dependent on the temperature of the inlet capillary. This effect is displayed in Figure 8a in mouse brain tissue, where imaging data show single ion images of several metabolites and lipids. The challenge in measuring accurate metabolite ratios is further explored by plotting the detected ion intensity of both lactate and glucose, as shown in Figure 8b. Comparing *m*/*z* 89.0239 (lactate) and *m/*z 179.0551 (glucose) in Figure 8a,b, we see that at 100 °C inlet temperature there is an intensity difference of close to 3 orders of magnitude. Conversely, at 255 °C, the ion intensities show only a slightly increased intensity for lactate over glucose. For example, the glucose/lactate ratio in cerebrospinal fluid (CSF) has been measured in the range of 1.1–3.4.^39^ While these values cannot be used to interpret the MSI data shown here due to the differing sample type and form, they do point to a potential route for further study and interpretation of inlet temperature, among other variables, in MSI. Future studies incorporating validation by, for example, LC-MS would provide an important ground truth.

**Figure 8:**
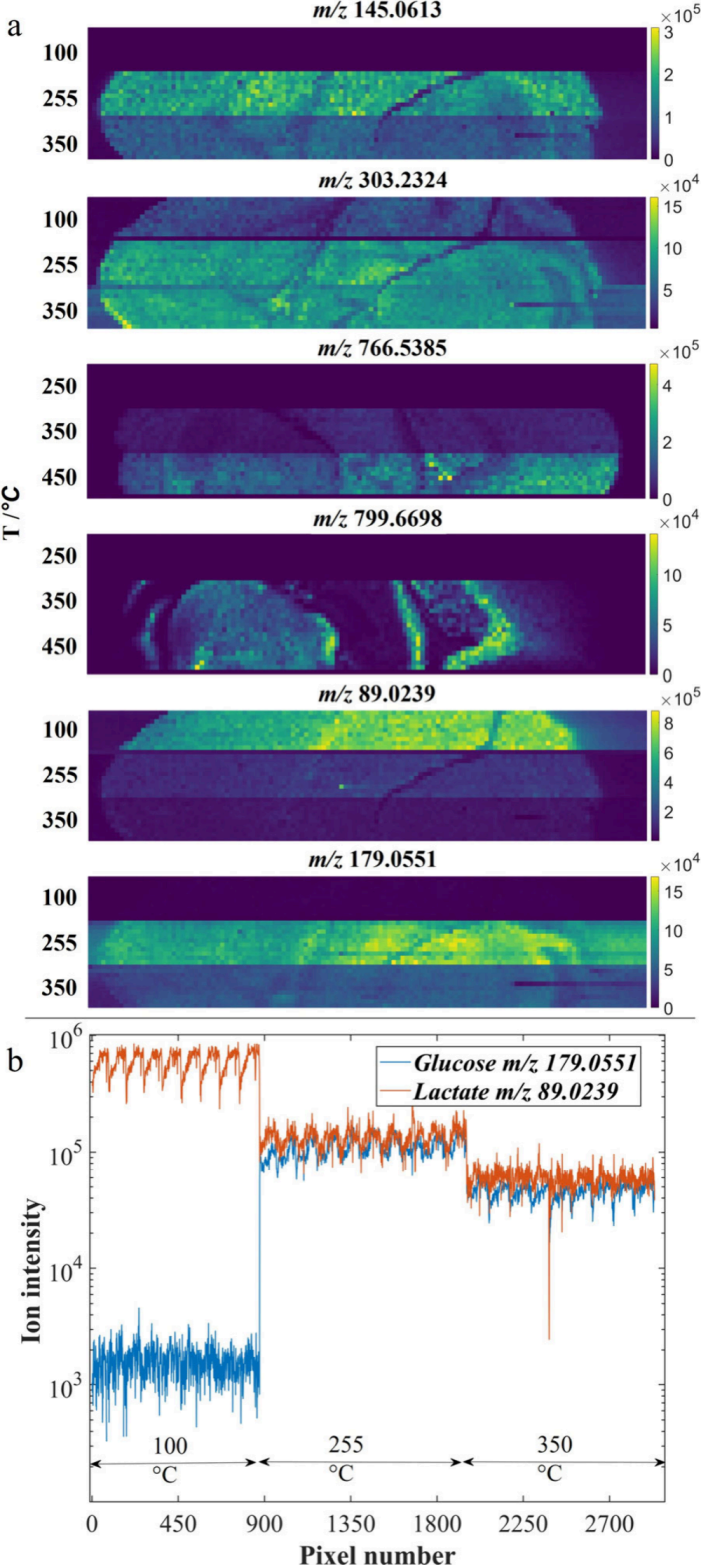
Single ion imaging of murine brain at various capillary inlet temperatures. (a) Heat map showing intensity of detected counts at labeled *m*/*z*. (b) Detected counts at each on-tissue pixel compared on log Y scale for ions assigned to glucose [M-H]^−^ and lactate [M-H]^−^, showing extreme ratio changes in detection intensity at different capillary temperatures.

A similar comparison made between the intensity of glucose *m*/*z* 179.0551 and arachidonic acid *m/*z 303.2324 would find approximately the same intensity of ion signals with an inlet temperature of 255 °C, but a clearly higher intensity of *m/*z 303.2324 over *m/*z 179.0551 at 350 °C, as inferred from the ion intensities in their single ion images (Figure 8a). Clear intensity differences at different temperatures can also be seen between PC­(15:0/20:4) *m/*z 766.5385 and SM­(d18:1/23:0) *m/*z 799.6698 for the temperatures 350 and 450 °C. Spatial distributions of the ions of interest are clearly visible in Figure 8; however, in each case represented, the intensity differences resulting from temperature change are visibly greater than the biological variation exhibited across the tissue itself. Ratio comparisons of metabolites in tissues could thus skew our interpretation of metabolic processes studied by MSI.

To maintain a sensible understanding of the species present when undertaking imaging, careful choice of inlet temperature is key for IR-PPI. In most cases, when comparisons of small metabolites are made, the recommended temperature of 250 °C should be sufficient, similarly for comparisons of lipids and the recommended temperature of 450 °C. However, if a particular ion comparison is of importance to a study, a study of standards at different temperatures and concentrations may be required to better inform interpretation of tissue imaging data.

Possible mechanistic factors related to inlet capillary temperature trends, such as those shown within this manuscript, were discussed in our previous DESI inlet capillary temperature study.^25^ In short, temperature in the capillary may influence fluid flow properties,^41^ ion charge transfer to capillary inner surface,^42^ droplet desolvation,^43^ and molecule degradation.^40^ For example, thermal degradation and transformation of, e.g., cholesterol, is known to occur to varying degrees in the range 120–220 °C^44^ and will likely accelerate at higher temperatures. The data presented here may, therefore, partially be explained by these degradation and transformation processes. A counterbalancing process which requires increased temperature would be thermally driven desolvation or declusting, thus competing mechanisms may give rise to the observed variable temperature behaviors seen within this and related studies.

In addition to our previous work^11^ demonstrating analysis of FFPE tissues with no sample preparation by IR-PPI, the study here further emphasizes the suitability of IR-PPI for FFPE tissue analysis. The lack of paraffin washing steps is a potentially highly valuable characteristic, as any further tissue treatment can result in delocalization and loss of soluble analytes and additional sources of variance. However, thorough comparison to MALDI and DESI MSI with washed tissue will still be a necessary and valuable step to assessing the suitability of these various modalities for metabolite coverage, distribution, and limit-of-detection in FFPE tissues.

## Conclusions

Throughout this paper, the effects in IR-PPI of inlet capillary temperature on the intensity of MSI ion signals, from tissue and background, have been presented. These changes are most pronounced when raising the inlet temperature above room temperature to 100 °C and above as very few identifiable tissue peaks are detected without inlet heating. It was found through multivariate analysis that the inlet temperature is a main driver of broad statistical changes in the detected mass spectra. Due to the likely ubiquity of inlet temperature effects for inlet mass spectrometry systems (DESI, LAESI, IRMALDESI, LA-PPI, nanoDESI etc.), it is highly relevant that practitioners evaluate the impact of temperature in their systems, be this for heated inlet capillaries or temperature settings in the MS inlet itself. A basic summary of the method used in the presented study is included in SI page S9 for reference to assist other practitioners.

Individual endogenous ions in tissue each show a single inlet temperature optimum for the highest detected ion current. These optima tend to be between 200 and 450 °C, and their detected intensities rise significantly above the background measurements with no laser interrogation. The fold change in intensity for ions of interest can be up to 17. This emphasizes the influence of temperature optimization in IR-PPI compared to prior inlet temperature work in DESI where the fold change varied by up to a value of 6.

Inlet temperature optima are broadly grouped by ion type. Small metabolites and amino acids were optimally observed between 200 and 300 °C, with outliers such as lactate (100 °C) and succinic acid (350 °C). Lipid signals were optimally observed between 400 and 500 °C, although CPA­(18:0/0:0) was optimal at 350 °C. These temperature ranges can inform untargeted studies, we include a table of ions of interest with their optimal inlet temperature for community use.

The effect of the substrate, so plume environment, did not appear to influence the optimal inlet temperature for glutamine. The temperature dependence of glutamine detection was consistent across three substrates and from endogenous glutamine in bovine brain homogenate, providing evidence that the changes in detection are rooted in the effect of inlet temperature on the specific analyte in question rather than the plume environment as a whole. Despite this, two ions detected across both FF and FFPE tissues (putative lactate and arachidonic acid ions) had optimal temperatures that differed by up to 100 °C. Further study with pure analytes may shed light on this discrepancy.

Comparison to similar prior work by DESI MSI indicates large differences between the behavior of IR-PPI and DESI, highlighting the larger impact of inlet temperature in IR-PPI. The lack of signal in IR-PPI at room temperature, in contrast to the DESI experiment, shows heated inlets are a necessity in IR-PPI. Not only this, but the ions that are mutually detected follow different temperature trends.

A difference in optimal temperature between two analytes can have a large impact on the interpretation of MSI results. An MSI comparison in mouse brain of the ion intensity of two important biological metabolites, lactate and glucose, at three different inlet temperatures, showed a change in the *m/z* intensity ratio that spanned close to 3 orders of magnitude. Caution is advised when using detected ion ratios in temperature dependent techniques to infer sample concentration ratios.

Future work consists of investigating the relationship between optimal inlet temperatures and the physicochemical properties of metabolites of interest. While a basic framework around the molecular class developed here can predict the approximate optimal conditions for broad classes of molecules, in time, the prediction of optimal inlet temperatures for targeted measurements may be possible.

## Supplementary Material



## Notes

Optical image of ablation tracks in tissue, mean mass spectra across two sample types, two *m*/*z* ranges and 6 temperatures, MVA results for FF pos ion and FFPE neg ion, and brief protocol for inlet temperature evaluation (PDF)
