# Supplementary material for: Effects of Inlet Capillary Temperature in Atmospheric-Pressure Infrared Laser-Ablation Plasma Postionization Mass Spectrometry
Source: J Am Soc Mass Spectrom. 2025 Oct 28;36(12):2633–46. doi: 10.1021/jasms.5c00243 (PMC12679636; doi:10.1021/jasms.5c00243)
Supplement: Supplementary file 1 [file js5c00243_si_001.pdf]

Supporting Information for: Effects of inlet capillary temperature in atmospheric-pressure infrared laser-ablation plasma post-ionization mass spectrometry

Lilian Ellis-Gibbings[1,&], Rory T. Steven[1,&,*], Alex J. Dexter[1], Josephine Bunch[1,2,*]

[1]National Physical Laboratory, Teddington, Middlesex, TW11 0LW. [2] Department of Metabolism, Digestion and Reproduction, London SW7 2AZ, UK. [&]Joint first authors. *Corresponding authors: rory.steven@npl.co.uk; Josephine.bunch@npl.co.uk

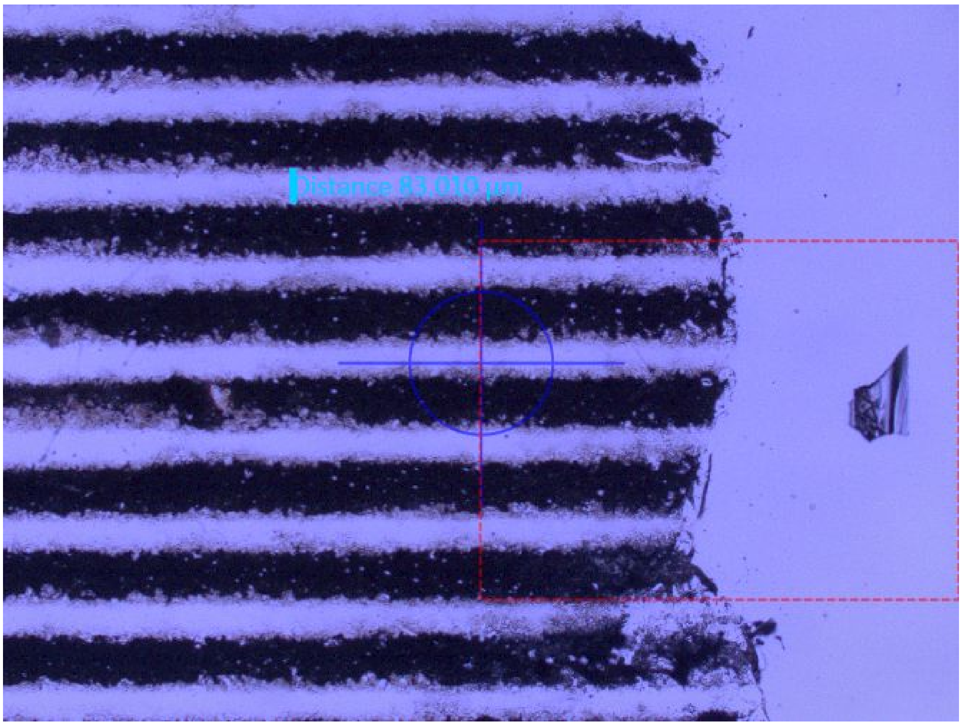

Figure S1. Microscope image showing ablation through liver tissue with same laser optical setup as employed within the presented study. Ablation track widths in the approximate range of 70 – 90 µm are evident.

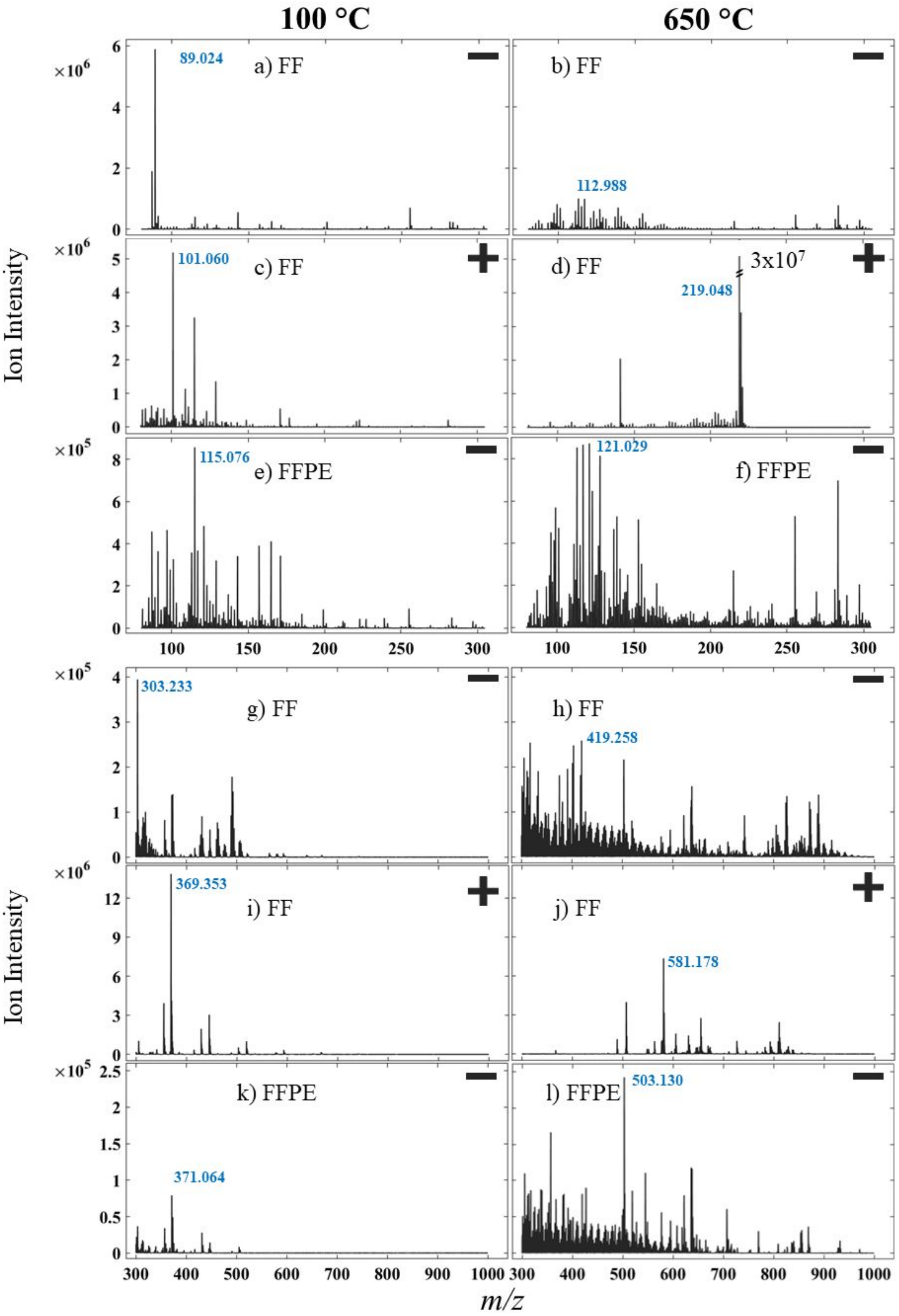

Figure S2. Mean mass spectra from IR PPI MSI highlighting the differences between 100 °C (left column) and 650 °C (right column) capillary inlet temperatures for a range of samples types, as follows. a/b/g/h) Fresh frozen BBH, negative ion mode. c/d/i/j) fresh frozen BBH, positive ion mode. e/f/k/l) FFPE mouse liver, negative ion mode. Mass ranges are 80-305 *m/z* (a-f) and 300-1000 *m/z* (g-l). Each spectrum is the mean from 20 pixels at the conditions specified. Basepeak *m/z* labelled.

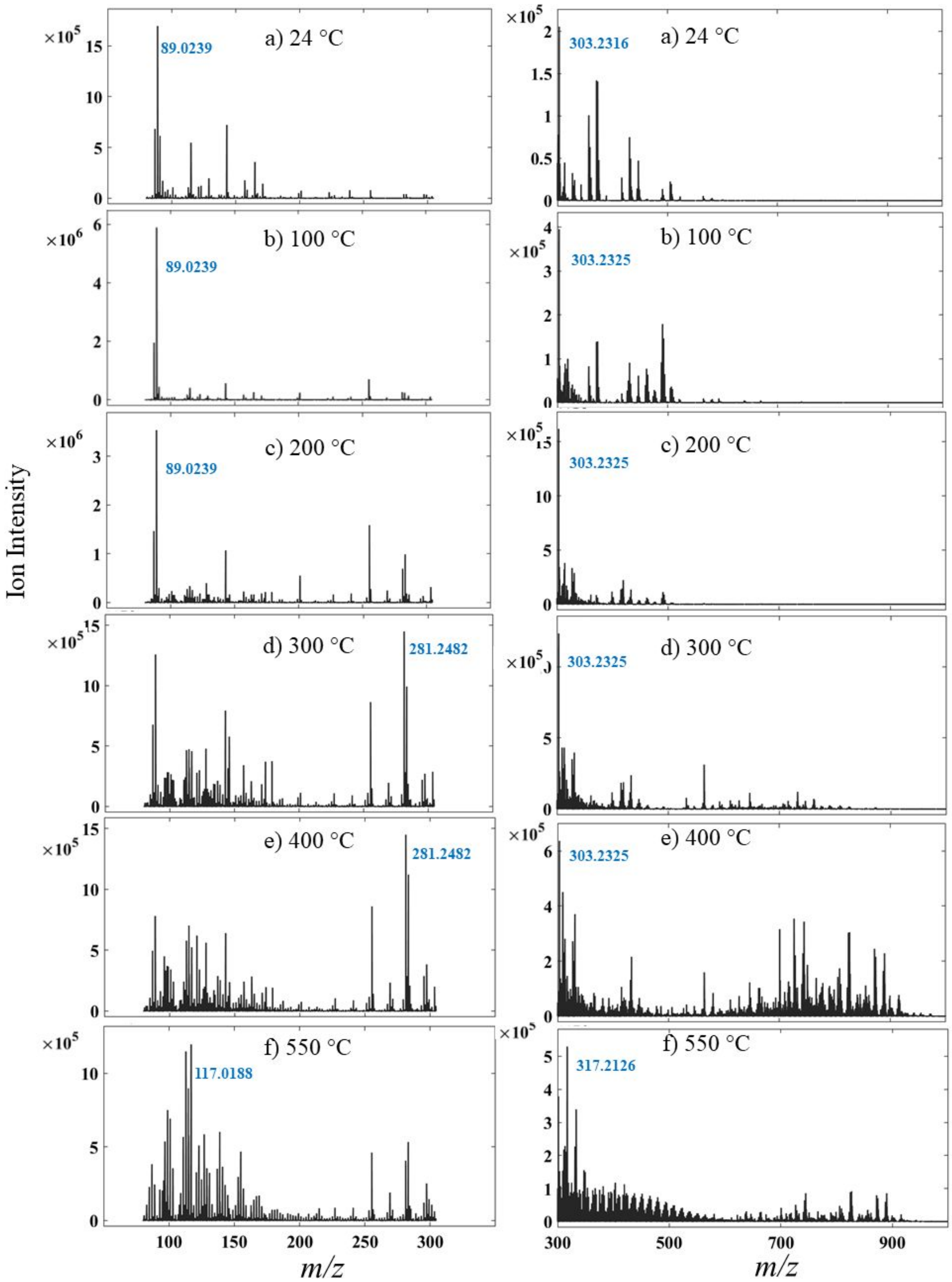

Figure S3. Mean mass spectra from IR PPI MSI highlighting the differences between across the capillary inlet temperature range of a) room temperature through to f) at 550 °C in fresh frozen BBH, negative ion mode. Mass ranges are 80-305 *m/*z (left column) and 300-1000 *m/*z (right column). Each spectrum is the mean from 20 pixels at the conditions specified. Basepeak *m/z* is labelled in all spectra.

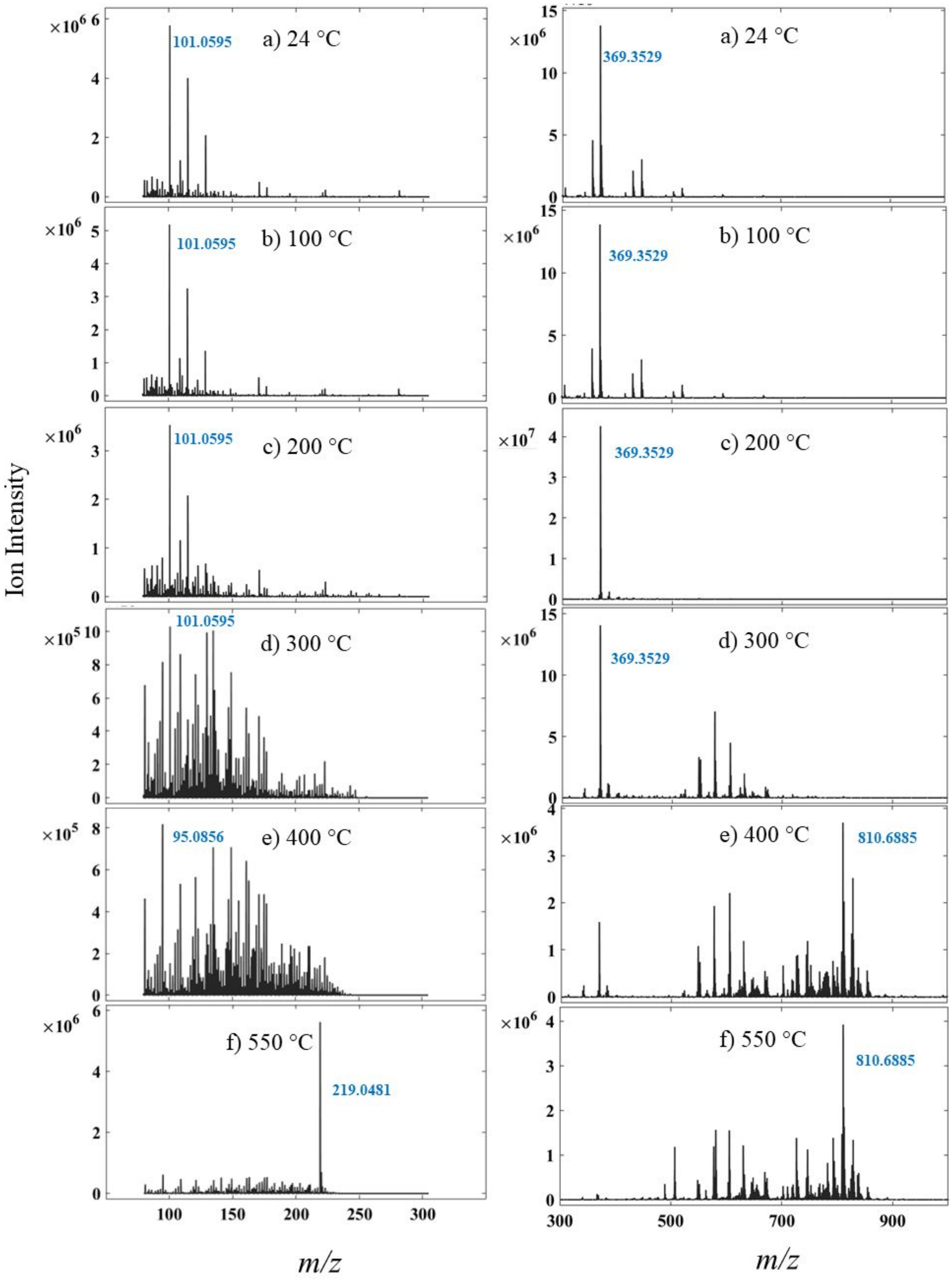

Figure S4. Mean mass spectra from IR PPI MSI highlighting the differences between across the capillary inlet temperature range for a) room temperature through to f) at 550 °C in fresh frozen BBH, positive ion mode. Mass ranges are 80-305 *m/*z (left column) and 300-1000 *m/*z (right column). Each spectrum is the mean from 20 pixels at the conditions specified. Basepeak *m/z* is labelled in all spectra.

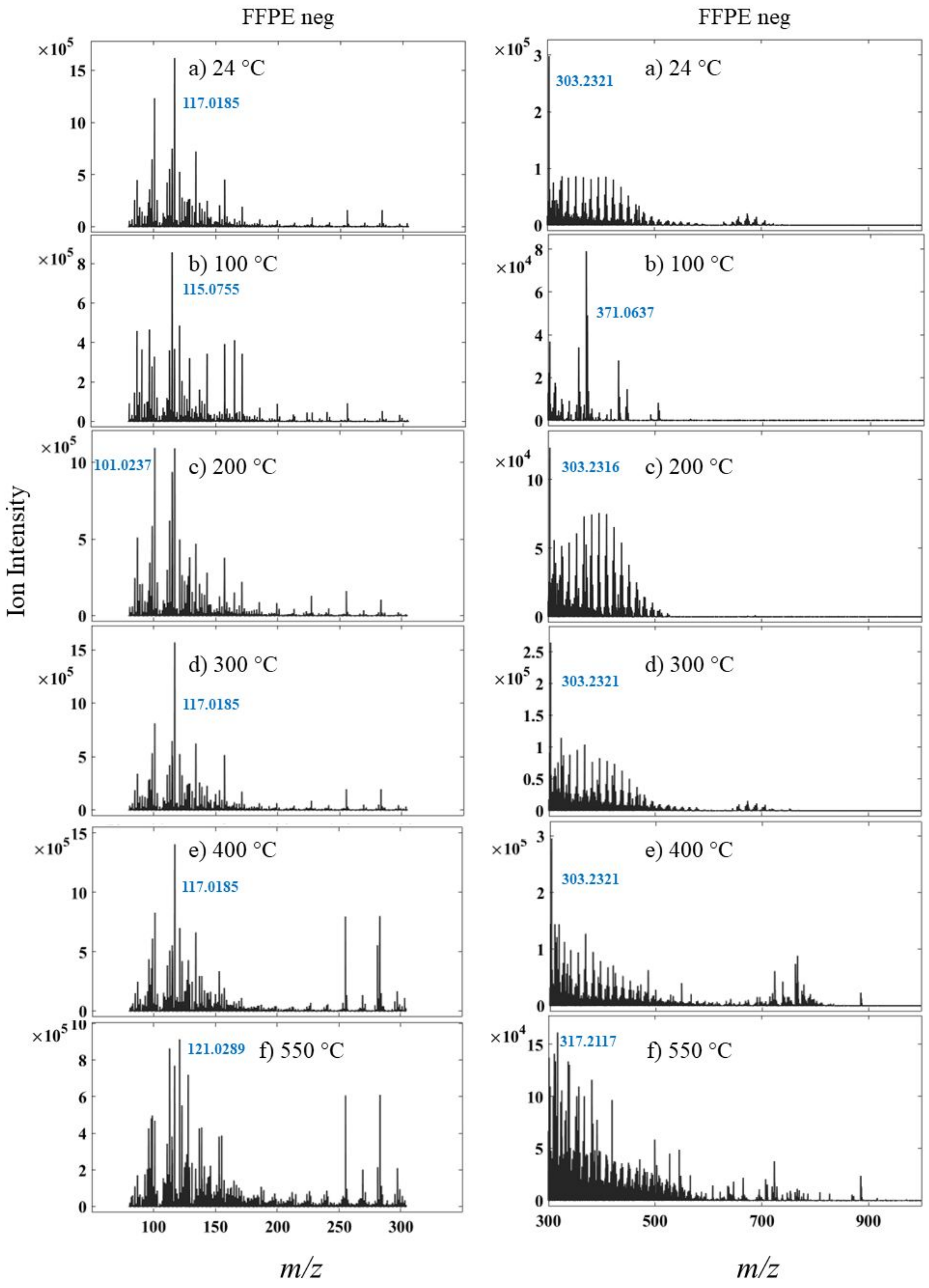

Figure S5. Mean mass spectra from IR PPI MSI highlighting the differences between across the capillary inlet temperature range for a) room temperature through to f) at 550 °C in FFPE mouse liver, negative ion mode. Mass ranges are 80-305 *m/*z (left column) and 300-1000 *m/*z (right column). Each spectrum is the mean from 20 pixels at the conditions specified. Basepeak *m/z* is labelled in all spectra.

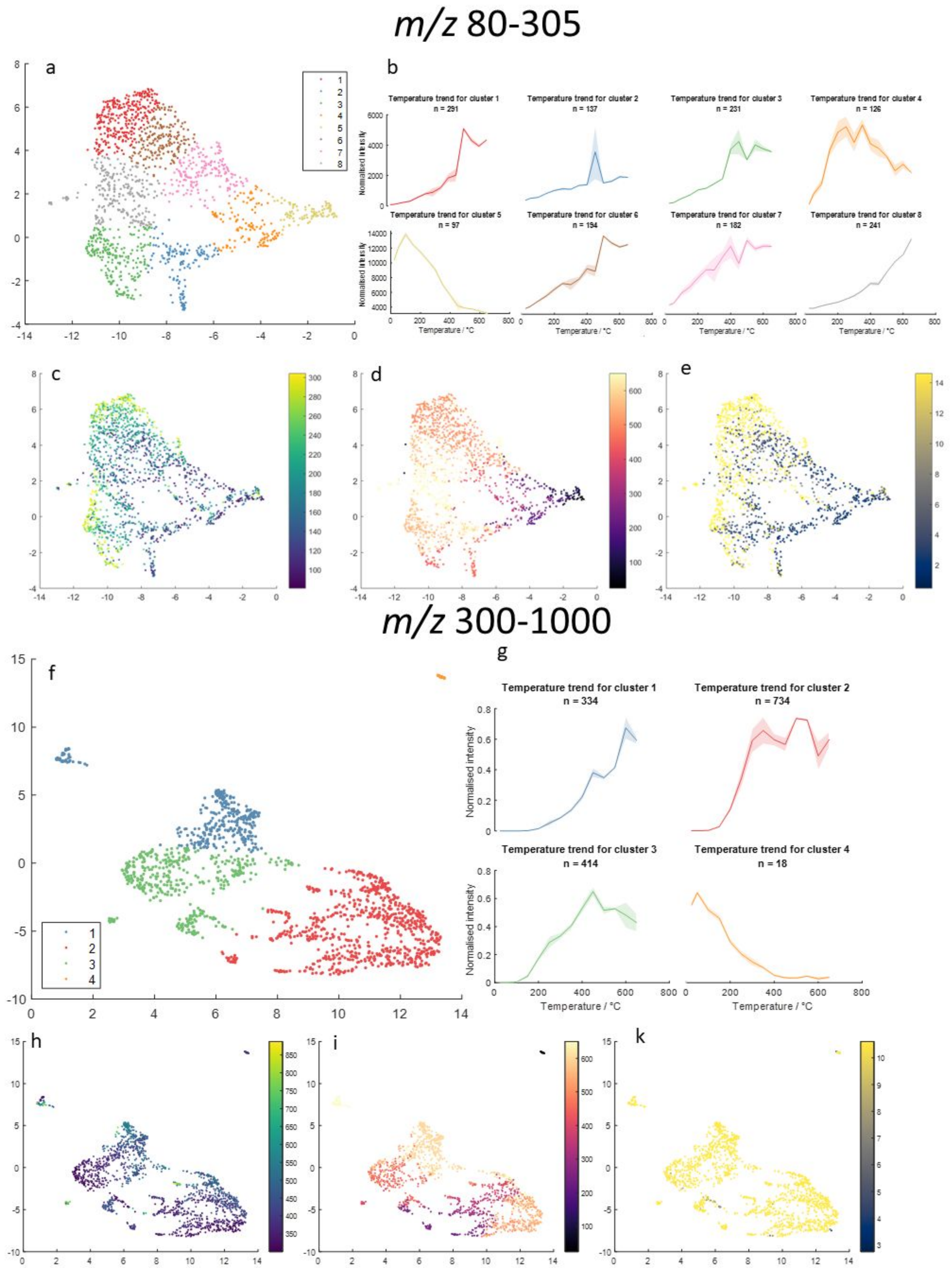

Figure S6. UMAP and k-means clustering across temperature trends. Negative ion mode, FFPE murine liver, measured across two mass ranges (m/z 80-305 a-e, and m/z 300-1000 f-j). UMAP embedding clustered by k-means clustering (a,f) alongside the associated average intensities across the temperature ranges per cluster (b, g). The UMAP embedding is also shown labelled according to key variables including m/z (c, h), optimal temperature (d, i), and log2(maximum intensity/minimum intensity) (e, j) to show the fold improvement by selecting an optimal inlet temperature.

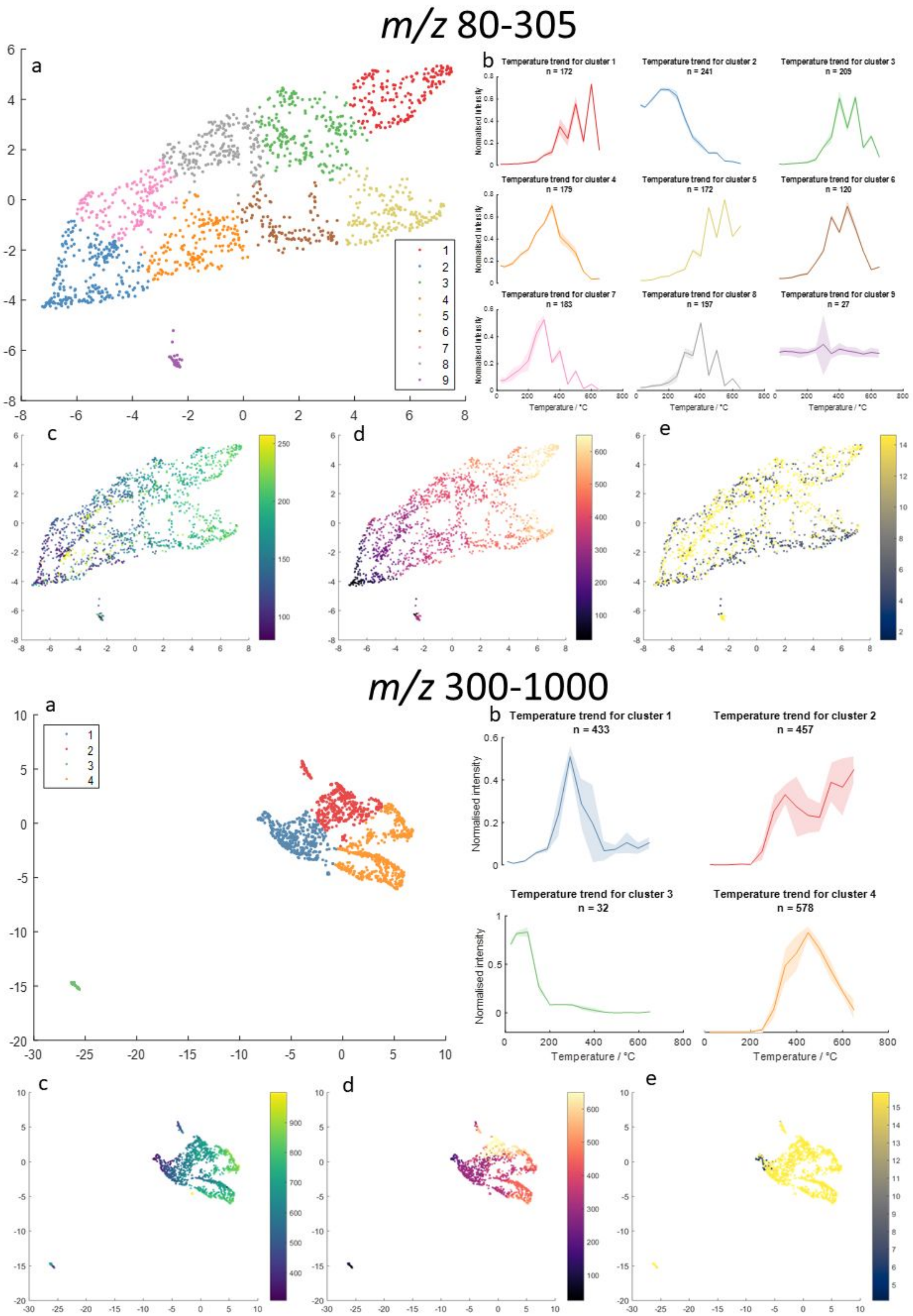

Figure S7. UMAP and k-means clustering across temperature trends. Positive ion mode, fresh frozen BBH, measured across two mass ranges (m/z 80-305 a-e, and m/z 300-1000 f-j). UMAP embedding clustered by k-means clustering (a,f) alongside the associated average intensities across the temperature ranges per cluster (b, g). The UMAP embedding is also shown labelled according to key variables including m/z (c, h), optimal temperature (d, i), and $\log_2$(maximum intensity/minimum intensity) (e, j) to show the fold improvement by selecting an optimal inlet temperature.

Simple summary of method used in this study for evaluating temperature effects in inlet MSI modalities

1. Section suitably homogenous tissue sample onto substrate (or potentially pipetted standard(s) on gelatine)
2. Set raster line length commensurate with sufficient number of pixels for subsequent statistical analysis
3. Analyse tissue along single line raster at fixed temperature
4. Collect background spectra where sampling probe is not interrogating tissue
5. Set new temperature and allow to stabilize
6. Repeat tissue sampling at new temperature on fresh area of tissue, avoiding oversampling
7. Repeat at appropriate intervals across temperature range, each on new (un-analysed) tissue region
8. Consider randomising or alternating temperatures to control for potential hysteresis effects
